# Circulating Tumor Cell Enumeration and Characterization in Metastatic Castration-Resistant Prostate Cancer Patients Treated with Cabazitaxel

**DOI:** 10.3390/cancers11081212

**Published:** 2019-08-20

**Authors:** Ingeborg E. de Kruijff, Anieta M. Sieuwerts, Wendy Onstenk, Jaco Kraan, Marcel Smid, Mai N. Van, Michelle van der Vlugt-Daane, Esther Oomen-de Hoop, Ron H.J. Mathijssen, Martijn P. Lolkema, Ronald de Wit, Paul Hamberg, Hielke J. Meulenbeld, Aart Beeker, Geert-Jan Creemers, John W.M. Martens, Stefan Sleijfer

**Affiliations:** 1 Department of Medical Oncology, Erasmus MC Cancer Institute, Erasmus University Medical Center, 3015 GD Rotterdam, The Netherlands; 2 Department of Medical Oncology, Franciscus Gasthuis & Vlietland, 3045 PM Rotterdam, The Netherlands; 3 Department of Medical Oncology, Gelre Ziekenhuizen, 7334 DZ Apeldoorn, The Netherlands; 4 Department of Medical Oncology, Spaarne Gasthuis, 2134 TM Hoofddorp, The Netherlands; 5 Department of Medical Oncology, Catharina Ziekenhuis, 5623 EJ Eindhoven, The Netherlands

**Keywords:** mCRPC, prostate cancer, circulating tumor cells, CTCs, biomarker

## Abstract

(1) Background: Markers identifying which patients with metastatic, castration-resistant prostate cancer (mCRPC) will benefit from cabazitaxel therapy are currently lacking. Therefore, the aim of this study was to identify markers associated with outcome to cabazitaxel therapy based on counts and gene expression profiles of circulating tumor cells (CTCs). (2) Methods: From 120 mCRPC patients, CellSearch enriched CTCs were obtained at baseline and after 6 weeks of cabazitaxel therapy. Furthermore, 91 genes associated with prostate cancer were measured in mRNA of these CTCs. (3) Results: In 114 mCRPC patients with an evaluable CTC count, the CTC count was independently associated with poor progression-free survival (PFS) and overall survival (OS) in multivariable analysis with other commonly used variables associated with outcome in mCRPC (age, prostate specific antigen (PSA), alkaline phosphatase, lactate dehydrogenase (LDH), albumin, hemoglobin), together with alkaline phosphatase and hemoglobin. A five-gene expression profile was generated to predict for outcome to cabazitaxel therapy. However, even though this signature was associated with OS in univariate analysis, this was not the case in the multivariate analysis for OS nor for PFS. (4) Conclusion: The established five-gene expression profile in CTCs was not independently associated with PFS nor OS. However, along with alkaline phosphatase and hemoglobin, CTC-count is independently associated with PFS and OS in mCRPC patients who are treated with cabazitaxel.

## 1. Introduction

The number of treatment options for metastatic castration-resistant prostate cancer (mCRPC) have increased rapidly in the last decade. Multiple treatment options are now available for mCRPC patients, including the chemotherapeutic agents docetaxel and cabazitaxel, the endocrine therapies abiraterone and enzalutamide, and options such as Radium-223 [1,2,3,4,5,6,7]. Therefore, biomarkers able to predict outcome to a particular treatment are necessary to select the right drug for the right patient at the right time. As the optimal treatment sequencing is currently unclear, one of the agents that would benefit from biomarkers for predicting which patients will benefit from that therapy is cabazitaxel, which is commonly given after progression to docetaxel in mCRPC patients.

The enumeration and characterization of circulating tumor cells (CTCs) hold great promise as predictive biomarkers for guiding optimal treatment sequencing. CTCs are cells that are shed from the tumor and/or metastatic lesions and can be isolated from the peripheral blood. In prostate cancer, the enumeration of CTCs is a strong independent prognostic factor for progression-free survival (PFS) and overall survival (OS) both before and during therapy, as shown in multiple studies [8,9,10,11]. However, the prognostic value for patients treated with cabazitaxel has only been analyzed in a small series of 56 patients [12].

Besides the enumeration of CTCs, characterization of these CTCs can help in predicting therapy response. One of the biomarkers that may carry predictive value is the androgen receptor (AR) splice variant 7 (AR-V7). Prior research has shown that AR-V7-positive patients benefit less from endocrine treatment than those who are AR-V7-negative [13], but that the AR-V7 status does not influence response to chemotherapy [14,15].

The advantage of CTCs is that they can be obtained in a minimally-invasive manner and mRNA of these cells can be isolated and analyzed. Therefore, gene expression profiles containing multiple genes can be measured in CTCs, which might be helpful in predicting outcome to therapies. 

Here, we aimed to identify markers associated with outcome to cabazitaxel therapy. To this end, we enumerated CTCs to confirm the prognostic value of CTC counts in mCRPC patients treated with cabazitaxel. Furthermore, we aimed to identify a gene expression profile determined in CTCs that is associated with outcome in mCRPC patients treated with cabazitaxel therapy.

## 2. Results

### 2.1. Patient Characteristics

As shown in Table 1, the median age of all 120 evaluable patients was 69 years (range 49–82 years). Only patients with a WHO performance score of 0 or 1 were included in the study. All patients had been previously treated with docetaxel. Only one patient received another line of chemotherapy besides docetaxel in a trial. In total, 36% of the patients had received anti-AR treatment (mainly abiraterone 23/43, 53%) for mCRPC before enrollment. Of the 114 patients with a known baseline CTC enumeration, 42 received anti-AR therapy and 72 did not. Of the patients who received anti-AR therapy, 14 (33%) patients had < 5 CTCs and 28 (67%) had ≥ 5 CTCs. In the 72 patients without endocrine pretreatment, 23 (32%) had < 5 CTCs and 49 (68%) had ≥ 5 CTCs (*p* = 1.00).

### 2.2. Circulating Tumor Cells at Baseline

In six of the 120 patients a baseline CTC enumeration could not be performed—three patients missed the baseline blood draw for enumeration and characterization of CTCs, one patient only missed the baseline blood draw for enumeration of CTCs, and two patients did not have enough blood in the CellSave tube to perform a reliable CTC enumeration. Therefore, in 114 patients a baseline CTC enumeration was performed. The median baseline CTC count was 15.5 (range 0–1025). In total, 37 patients had < 5 CTCs at baseline and the remaining 77 had ≥ 5 CTCs. When looking at the patient characteristics, only WHO performance status, LDH and alkaline phosphatase were significantly different between the patients with < 5 and ≥ 5 CTCs (*p* = 0.045, *p* = 0.001 and *p* < 0.001, respectively) (see Table 1).

**Figure 1:**
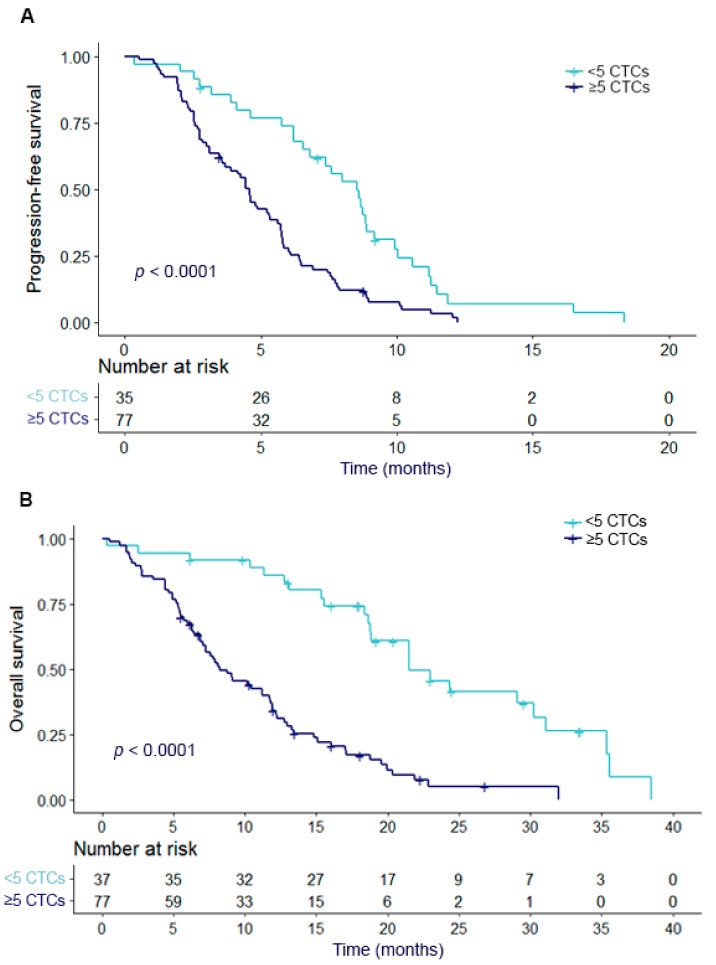
PFS and OS in relation to dichotomized CTC count at baseline. Kaplan Meier curves of (**A**) progression-free survival (PFS) and (**B**) overall survival (OS) in relation to circulating tumor cell (CTC) count at baseline. CTC counts are divided into two categories of < 5 CTCs and ≥ 5 CTCs.

Patients with <5 CTCs before start of cabazitaxel therapy had a significantly better PFS and OS as compared to patients with ≥ 5 CTCs at baseline (both *p* < 0.001) (Figure 1). The median PFS in the entire cohort was 5.3 months. Patients with < 5 CTCs had a median PFS of 8.0 months, while this was 4.4 months for patients with ≥ 5 CTCs (*p <* 0.001). The median OS was 11.8 months in all patients (*n* = 114), 18.9 months in the patients with < 5 CTCs, and 7.9 months for patients with ≥ 5 CTCs (*p <* 0.001). 

### 2.3. Circulating Tumor Cell Dynamics at Baseline and Follow-Up

A matched baseline and follow-up (at 6 weeks) CTC enumeration was available for 95 patients. These patients were divided into four groups: group 1 contained patients with < 5 CTCs at baseline who remained < 5 CTCs during follow-up (*n* = 24); group 2 had ≥ 5 CTCs at baseline but had < 5 CTCs during therapy (*n* = 19); group 3 had < 5 CTCs at baseline and ≥ 5 CTCs during therapy (*n* = 5); and group 4 were patients who had ≥ 5 CTCs both at baseline and during therapy (*n* = 47). 

**Figure 2:**
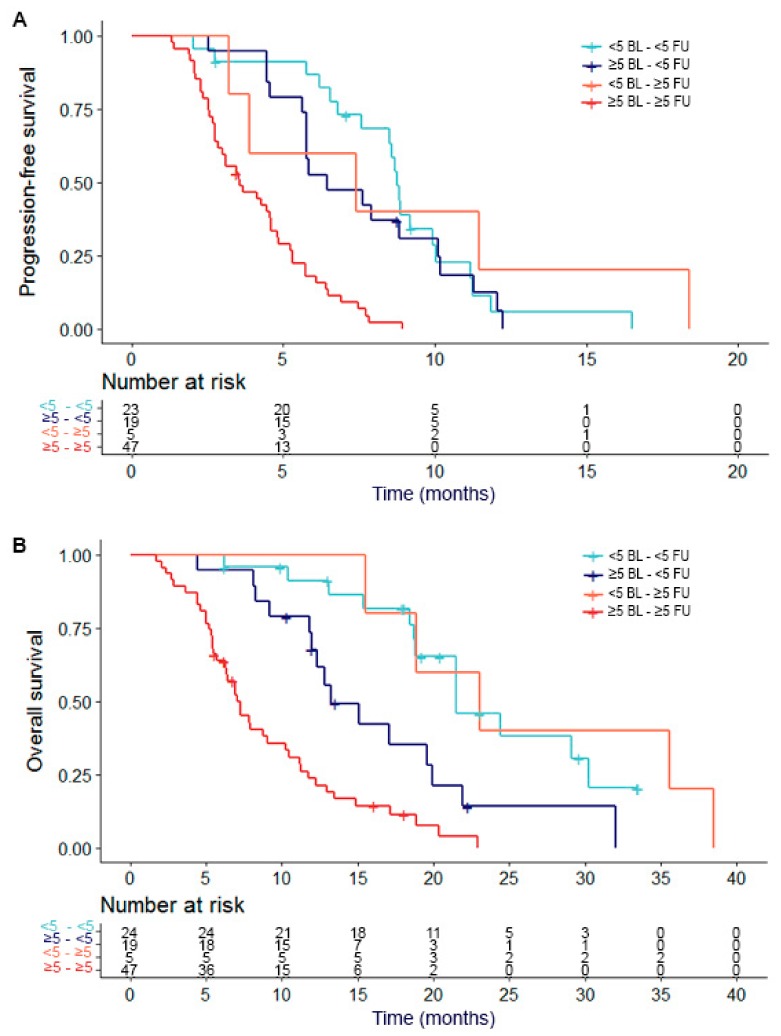
PFS and OS in relation to CTC groups. Kaplan Meier curves of (**A**) progression-free survival (PFS) and (**B**) overall survival (OS) in relation to circulating tumor cell (CTC) count. CTC counts are divided into four categories: (1) <5 CTCs at baseline and <5 CTCs during treatment, (2) ≥5 CTCs at baseline and <5 CTCs during treatment, (3) <5 CTCs at baseline and ≥5 CTCs during treatment and (4) ≥5 CTCs at baseline and ≥5 CTCs during treatment.

Median PFS for group 1 was 8.7 months, while it was 6.4 months for group 2, 7.4 months for group 3, and 3.5 months for group 4. There was a significant difference between group 4 and group 1 (*p* < 0.001), group 2 (*p* < 0.001), and group 3 (*p* = 0.032). Between the other groups, no significant difference was found. Median OS for group 1 was 19.0 months, while it was 12.8 months for group 2, 23.0 months for group 3, and 6.9 months for group 4. For OS, there was also a difference between group 4 and group 1 (*p* < 0.001), group 2 (*p* = 0.003), and group 3 (*p* = 0.003). Also, a difference between group 1 and 2 was found (*p* = 0.016). In Figure 2, a Kaplan–Meier plot for PFS and OS of the different groups of patients is shown. 

### 2.4. Gene Expression Profiles of CTC-Specific Genes

To evaluate the predictive value of gene expression levels for therapy outcome for patients treated with cabazitaxel, 91 genes were measured (see Supplementary Table S1a). Of these genes, 34 had a significantly higher expression in CTC samples compared to samples from healthy blood donors (HBDs) (with a false discovery rate (FDR) of 5%). These genes are listed in Supplementary Table S1b. Hierarchical clustering of the 34 CTC-specific genes in all baseline samples with available gene expression data (*n* = 63) divided the samples into two groups (Supplementary Figure S1). The median CTC count in the first group was 19, while this was 154 in the second group (*p* < 0.001). However, when these two subgroups were visualized in a Kaplan–Meier plot for PFS and OS and tested with the Log-Rank test, there was no difference in PFS or OS between the groups (see Supplementary Figure S2).

### 2.5. Gene Expression Profiles in Relation to Outcome to Cabazitaxel Therapy 

The 34 CTC-specific genes were also analyzed to determine a gene expression panel that predicts outcome to cabazitaxel therapy. In the 63 baseline samples, five genes were identified with the highest discriminatory power to predict PFS on cabazitaxel therapy. These genes were *AGR2, FOXA1, DKK1, FAT1* and *TMPRSS2*. These were also the genes that were significantly associated with prolonged PFS in univariate Cox regression analysis (see Supplementary Table S2). The patients were divided into two groups using a median split of the predicted risk score, with 32 patients having a high predicted risk score and 31 patients having a low predicted risk score. However, these groups did not show statistically different survival curves for PFS (*p* = 0.27). When we used this estimated risk score for OS, there was a difference in outcome between the high and low risk groups (*p* = 0.039). Supplementary Figure S3 shows the Kaplan Meier curves of both PFS and OS in relation to the high and low risk groups.

### 2.6. Cox Regression Analysis

As the primary objective of this study was to identify the most powerful markers for outcome to cabazitaxel therapy, all available variables (baseline CTC count, age, PSA, alkaline phosphatase, lactate dehydrogenase (LDH), albumin, hemoglobin and the 5-gene profile) were analyzed in uni- and multivariate Cox regression models for both PFS and OS in the 63 patients for which all of these data were available. For PFS, univariate Cox regression analyses showed a significant association with both CTC count and hemoglobin. In multivariate regression analysis, both CTC count (HR 1.001, 95% CI 1.00–1.00, *p* = 0.023) and hemoglobin (HR 0.731, 95% CI 0.56–0.95, *p* = 0.020) remained independent prognostic factors for PFS (see Table 2A). For OS, five variables were significant in univariate Cox Regression analysis: the 5-gene profile, CTC count, alkaline phosphatase, LDH and hemoglobin. In multivariate analysis, only CTC count (HR 1.002, 95% CI 1.00–1.00, *p* = 0.009) and hemoglobin (HR 0.642, 95% CI 0.48–0.85, *p* = 0.002) remained significant independent factors for OS (see Table 2B).

When looking at the baseline variables without the 5-gene expression profile, 114 patients had all of these data available. Baseline CTC count, alkaline phosphatase, LDH and hemoglobin were significant in univariate analysis for PFS. In multivariate analysis, CTC count (HR 1.002, 95% CI 1.00–1.00, *p* < 0.001), alkaline phosphatase (HR 1.002, 95% CI 1.00–1.00, *p* = 0.014), and hemoglobin (HR 0.749, 95% CI 0.61–0.92, *p* = 0.005) remained independent prognostic factors for PFS (see Table 3A). For OS, CTC count, alkaline phosphatase, LDH, albumin and hemoglobin were significant in univariate analysis. In multivariate analysis, only CTC count (HR 1.002, 95% CI 1.00–1.00, *p* < 0.001), alkaline phosphatase (HR 1.002, 95% CI 1.00–1.00, *p* = 0.006), and hemoglobin (HR 0.591, 95% CI 0.47–0.74, *p* < 0.001) remained independent prognostic factors (see Table 3B).

## 3. Discussion

Our data confirms the strong prognostic value of CTCs in patients treated with cabazitaxel. We found the CTC count to be independently associated with outcome in mCRPC patients who were treated with cabazitaxel in the second line of chemotherapy, besides the well-known prognostic parameters hemoglobin and alkaline phosphatase. To the best of our knowledge, this is the largest study investigating the association between CTC numbers and outcome to cabazitaxel treatment. Climent et al. have assessed baseline CTC numbers in 56 patients receiving cabazitaxel therapy. They also found an association between CTC count and PFS, however they did not find a statistically significant association between CTC count and OS [12]. 

Conversions between the favorable group (< 5 CTCs) and unfavorable group (≥ 5 CTCs) have been described in mCRPC patients, however not yet in a large set of cabazitaxel treated patients. Literature has shown for both metastatic breast and prostate cancer that patients who are in the favorable group at baseline and follow-up have the best prognosis, while patients who are in the unfavorable group at baseline and follow-up have the worst prognosis [8,16]. In these studies, mCRPC patients who switched from ≥ 5 CTCs at baseline to < 5 CTCs at follow-up had a better prognosis than patients who had < 5 CTCs at baseline but increased to ≥ 5 CTCs at follow-up. In the current study, we looked at baseline CTC count and CTC count after six weeks of therapy instead of after therapy failure, which was done in the previous described study [8]. We chose the six-week time point as our aim was to detect early progression in our patients. This would allow patients to switch therapies in an early stage of therapy. Our data showed that after dividing our patients into four groups depending on CTC count before and during therapy, patients with an unfavorable CTC count at baseline and during treatment indeed had the worst prognoses. However, the difference between the other 3 groups was less clear. The relatively small sample size of these subgroups (especially group 3 (< 5 CTCs to ≥ 5 CTCs) contained only 5 patients) limits the power of this analysis, so based on our data no firm conclusion can be drawn for CTC conversions during therapy. Preferably, this analysis should be repeated with more patients and with CTC count assessed at progression of disease.

In multivariable analysis for both PFS and OS, CTC count, alkaline phosphatase and hemoglobin were independent prognostic factors. Hemoglobin and alkaline phosphatase are known prognostic factors in mCRPC [17]. Patients with lower hemoglobin levels (< 12 g/dL) have shorter OS, while for CTC count and alkaline phosphatase the relation is inversed. Alkaline phosphatase originates mainly from bone and liver, with 40–50% coming from bone in adults [18]. Since 90% of the mCRPC patients have radiologic evidence of bone metastasis [3], alkaline phosphatase is an important biomarker in prostate cancer. For cabazitaxel, no direct effect on alkaline phosphatase is known, only indirect when the tumor burden decreases, alkaline phosphatase levels also decrease [19]. Also for hemoglobin no direct effect of cabazitaxel is known. Both alkaline phosphatase and hemoglobin can be influenced by tumor burden, but also by a patient’s underlying condition. CTCs are the only factor that reflect the tumor burden directly. However, all of these markers together can help physicians in deciding which patients should receive cabazitaxel treatment at that moment.

In addition to traditional clinical parameters and CTC counts, we also assessed the clinical value of a CTC derived gene expression profile to gain more insight into the biology of the tumors. Therefore, 91 genes with proven involvement in prostate cancer were measured in mRNA from the CTC enriched samples. Of these genes, 34 genes had higher expression in the CTCs than in the leukocyte background and were used for further analysis. Using these 34 genes to cluster the 63 samples for which baseline gene expression data were available, the samples were clearly divided into two groups, which showed a significant difference in CTC count. The cluster could be biased by CTC count, however, we do observe clear variation in gene expression beyond sheer CTC count, indicating that the differences are probably not just driven by numbers of CTCs.

The five genes from the gene expression signature with the highest discriminatory power to predict PFS on cabazitaxel were *AGR2, FOXA1, DKK1, FAT1* and *TMPRSS2*. All genes used for the gene expression panel were selected based on expression and clinical relevance in prostate cancer. Looking at these five genes, *AGR2* is a CTC-specific gene in metastatic breast and colorectal cancer that has been associated with poor outcome in prostate cancer patients [20,21,22,23]. *FOXA1* is a key component of the AR transcriptional complex and overexpression leads to enrichment of the PTEN and WNT signaling pathway [24]. *DKK1* is a WNT signaling inhibitor and has been associated with poor survival in prostate cancer patients [25]. *FAT1* is a tumor suppressor gene that affects the WNT signaling pathway [26] and *TMPRSS2* is an AR-regulated gene that is involved in a gene fusion (*TMPRSS2-ERG*) in prostate cancer [27]. Prostate cancers with a positive *TMPRSS2-ERG* fusion show increased WNT signaling [28]. In summary, these genes are especially involved in the AR and WNT signaling pathways. While the role of the AR pathway has been well-established in prostate cancer, the role of the WNT signaling pathway is less clear. However, changes in the WNT signaling pathway have been described in prostate cancer, especially in the development of mCRPC. The genes presented here emphasize the need to investigate the role of the WNT pathway in mCRPC and eventually look at the possibility of using WNT modulators in mCRPC patients [29,30].

The gene expression signature of five genes to predict outcome on cabazitaxel therapy showed no significant difference in PFS. Reviewing the Kaplan–Meier curve, a difference in the first 7 months of treatment is visible. However, after that moment, the number of patients is too small to make firm conclusions. For OS, there was a significant difference based on this 5-gene profile, however, in multivariate analysis this was not an independent prognostic factor. Literature shows several studies with gene expression profiles determined in peripheral blood [31,32,33,34,35,36] or on CTCs [37,38,39,40,41,42,43] that are associated with outcome in patients with metastatic prostate cancer. Especially the studies of Wang et al. [36], Olmos et al. [34], and Ross and al. [35], in which gene expression profiles were established in whole blood of mCRPC patients, showed prognostic value of the established gene profiles in an independent training and validation set. However, all of the studies mentioned above still lack prospective validation. Also, none of these studies assessed a gene expression profile that purely predicts response to cabazitaxel. Despite the lack of clinical value of the approach we described here, we still feel that assessing the clinical value of gene expression profiles established in CTCs is worth pursuing. CTCs, as enumerated by the CellSearch system, which probably does not isolate the CTCs with a more mesenchymal phenotype [44], do have strong prognostic power, as shown here and in many other studies, and therefore represent a biologically relevant population of cells. A limitation of CTC isolation with the CellSearch system is the background of leukocytes present after enrichment of CTCs, however, there is less background present than after isolation of peripheral blood mononuclear cells (PBMCs). This background severely limits the number of genes that can be evaluated, since only genes can be evaluated that are solely expressed in CTCs and not in leukocytes. Using techniques that enable the isolation of pure fractions of CTCs would increase the number of genes that can be evaluated for prognostic and predictive relevance and also gives the opportunity to assess heterogeneity between CTCs within one patient, and therefore deserves further study. 

A limitation of this study was the number of patients that were available for analysis. For CTC comparison analysis, 95 matched CTC samples were available. However, when dividing these patients into different subgroups, some subgroups were too small to make firm conclusions. Also, for baseline gene expression samples only 63 samples were available, of which almost all patients had ≥ 5 CTCs, which made it difficult to make comparisons in this group. Furthermore, this group mostly contained patients with worse prognoses based on their CTC count. 

## 4. Materials and Methods 

### 4.1. Patients

**Figure 3:**
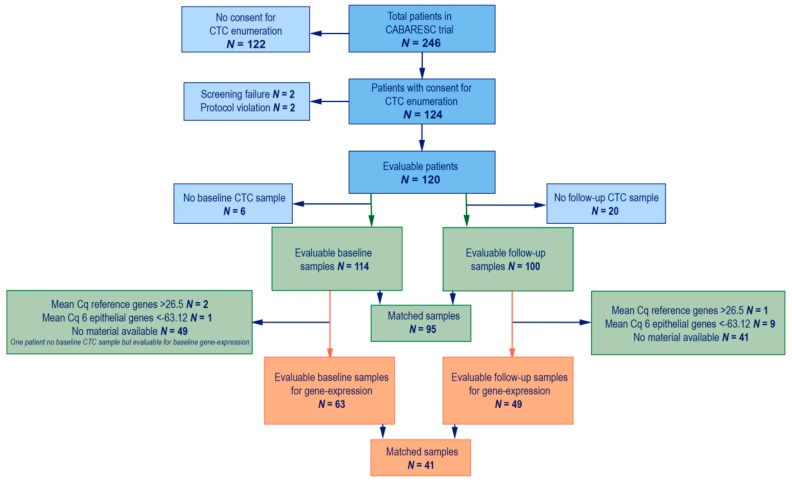
Patient inclusion. Flowchart of patient inclusion. In blue all patients who are included in the CABARESC study and the side CTC enumeration study. In green the patients included for this manuscript with available CTC enumeration. In Orange the patients included for this manuscript with available gene expression data.

The mCRPC patients starting with a new line of cabazitaxel treatment were selected from a prospective multicenter trial (CABARESC trial, NTR2991). In this study, mCRPC patients who progressed after docetaxel treatment for CRPC were included and randomized to receive 25 mg/m^2^ of cabazitaxel with or without budesonide treatment [45]. Treatment with cabazitaxel continued until progression of disease or unacceptable toxicity with a maximum of ten cycles. The primary objective of CABARESC was to evaluate the effects of budesonide on cabazitaxel-induced diarrhea [45]. As a side study, blood was drawn for CTC enumeration and characterization in this trial. If a patient provided consent to participate in this side study, blood was drawn before the start of cabazitaxel treatment and before the third cycle of cabazitaxel. In total, 246 mCRPC patients were included, of whom 124 provided consent for CTC enumeration and characterization. Of these 124 patients, four patients were excluded due to protocol violation (*n* = 2) and screen failure (*n* = 2). Therefore, a total of 120 patients were available for this study (see Figure 3). The study was approved by the Erasmus MC and local Institutional Review Boards (METC 11–324). All patients provided written informed consent. Clinical data were collected from all patients.

### 4.2. CTC Enumeration and mRNA Isolation

For CTC enumeration 7.5 mL blood was collected in a CellSave tube and for characterization 7.5 mL blood was collected in an EDTA tube before start and during cabazitaxel treatment. Blood was processed with the CellSearch system (CellSearch enumeration kit and CellSearch profile kit, Menarini-Silicon Biosystems, Huntington Valley, PA, USA). For enumeration, blood was processed within 96 hours, while for characterization blood was processed within 24 hours. After CTC enrichment, mRNA was isolated with the AllPrep DNA/RNA Micro Kit (Qiagen, Germantown, MD, USA). Thereafter, cDNA was generated, which was pre-amplified for the targets of interest and real time amplified (RT-qPCR) using Taqman Gene Expression Assays (Applied Biosystems, Carlsbad, CA). For a more detailed description, see previously published papers [22,46].

A baseline CTC sample was collected in 114 of the 120 patients. For six patients a baseline CellSave tube was not drawn, and therefore CTCs could not be enumerated. For 100 patients a follow-up sample was collected after six weeks. For the gene expression assays, material from 66 baseline samples and 59 follow-up samples was available. However, three baseline samples and ten follow-up samples were excluded due to insufficient cDNA quality or quantity (see below). Therefore, 63 baseline samples and 49 follow-up samples were included for gene expression analysis, which contained 41 matched samples (see Figure 3).

### 4.3. Sample Processing and Normalization

A gene expression profile of 147 genes was developed based on expression and clinical relevance in prostate cancer, as derived from literature. Since a background of leukocytes remains after CTC isolation with the CellSearch System, only genes with higher expression in prostate cancer than in leukocytes were selected. Therefore, all genes were reviewed for expression in leukocytes and in prostate cancer (SAGE Genie Database of the Cancer Genome Anatomy Project (http://cgap.nci.nih.gov/SAGE/AnatomicViewer)). The 91 genes with the lowest leukocyte expression or highest upregulation in prostate cancer (> 10×) were selected. Besides these 91 genes, three reference genes (*GUSB*, *HMBS*, and *HPRT1*) were added to determine the quality of the sample, along with a negative control (H_2_O) and a marker for leukocyte contamination (PTPRC, Protein Tyrosine Phosphatase Receptor type C). Samples with an average reference gene signal of ΔC*_q_* > 26.5 were considered to be of insufficient cDNA quantity and/or quality, and were therefore excluded (*n* = 3).

For each of the 91 genes, the ΔC*_q_* of 15 CellSearch enriched healthy blood donor (HBD) samples was determined. A CTC sample was considered positive for a given gene if the C*_q_* value was at least one standard deviation higher (+1SD) than the mean ΔC*_q_* of the 15 HBDs. If the expression of a gene was below this cut-off, it was considered not detectable and given the value of the mean ΔC*_q_* of the 15 HBDs.

To further correct for the leukocyte background and ensure the presence of a CTC-derived signal, an epithelial profile was calculated for every sample. Previously, a 12-gene signal was identified that was associated with epithelial tumor load in breast cancer patients [22]. The gene expression profile used here for prostate cancer contained 6 of these 12 genes. Therefore, we correlated these 6 genes (*AGR2, FOXA1, EPCAM, IGFBP5, LAD1* and *S100A16*) from our prostate panel with the 12 genes from the epithelial signal, which was measured in CTCs of 910 breast cancer patient samples and 20 HBD samples. For these 930 CTC samples, the ΔC*_q_* sum of these 12 genes resulted in a cut-off of -131 to identify the presence of at least one CTC in a sample. After correction for the slightly different HBD-corrected cut-off in this prostate cancer panel than in the breast cancer panel, the cut-off used here based on the ΔC*_q_* of six genes to identify a positive epithelial signal, and therefore presence of at least one CTC, was -63.12 (see Supplementary Figure S4). Based on this epithelial cut-off, 10 samples (1 baseline and 9 follow-up samples) were excluded for supposedly not containing any CTC-derived signal. Therefore, a total of 63 baseline samples and 49 follow-up samples were available for comparison of the gene expression profiles at baseline and after 6 weeks of therapy.

### 4.4. Statistical Analysis

Patient characteristics were compared between those with < 5 CTCs and those with ≥ 5 CTCs by means of chi-square tests for categorical variables and the Mann–Whitney U test for continuous variables. The cut off of 5 CTCs was chosen as this has been proven to be prognostic in patients with mCRPC in several independent studies [8,47]. Progression free survival (PFS) was defined as time between start of treatment and progression of disease, and overall survival (OS) was defined as time from start of treatment until death by any cause. CTC count was determined both at baseline and follow-up. Besides studying the effect of baseline CTC count as a marker for the survival outcomes, the four possible groups of CTC count development (i.e., CTC count remaining smaller than 5 or ≥ 5, CTC count increasing from < 5 to ≥ 5, or the other way around) were also studied. Furthermore, a gene expression profile was developed and related to survival outcomes. For all these variables the relation with PFS and OS was studied by means of log-rank tests and visualized with Kaplan–Meier plots. Furthermore, univariate and multivariate Cox proportional hazards analyses were performed to quantify the relationships further. For the multivariate analyses, a stepwise backward procedure was followed including all available variables, and a threshold of *p* < 0.05 was applied for the selection of variables to remain in the model.

In order to select the genes from the gene expression panel that were CTC-specific, we compared expression levels per gene in the 112 patient CTC samples and in 15 HBD samples with the Mann–Whitney U test. To correct for multiple testing, a FDR of 5% was applied. Gene expression data of the 63 baseline samples were compared with the Survival Risk Prediction (SRP) tool from Biometric Research Branch ArrayTools (BRB-ArrayTools, http://linus.nci.nih.gov/BRB-ArrayTools.html), using a *p*-value of < 0.05. For hierarchical clustering, both the genes and the samples were normalized, the genes were centered, and average uncentered clustering was performed using Cluster 3.0 and visualized with Java Treeview version 1.6. All computations were performed using R (version 3.4.1) and all reported *p*-values are two-sided (unless stated otherwise).

## 5. Conclusions

These data show that characterization of CTCs as of yet did not hold sufficient power to distinguish outcome to cabazitaxel treatment. However, our data confirms the strong, independent prognostic value of the CTCs for both PFS and OS in mCRPC patients that are treated with cabazitaxel in the second line, together with alkaline phosphatase and hemoglobin.

## Figures and Tables

**Table 1:** Patient characteristics.

Category	N	%	< 5 CTC	≥ 5 CTC	*p*-Value
Age					0.968
< 65 years	31	26	10	19	
≥ 65 years	89	74	27	58	
WHO performance score					0.045
0	61	51	24	33	
1	59	49	13	44	
Type of castration					1.000
Surgical	15	13	5	10	
LHRH agonist	105	88	32	67	
Prior chemotherapy lines					
1 (only docetaxel)	119	99	37	76	
2	1	1	0	1	
Prior antiandrogen therapy for mCRPC *					0.326
Abiraterone	23	19	10	12	
Enzalutamide	9	8	2	7	
Orteronel	12	10	3	9	
None	77	64	23	49	
Baseline chemistry	Median	Range	Median (range)	Median (range)	
LDH U/L (*n* = 118)	312	141–1843	237.5 (151–531)	353 (141–1843)	0.001
ALP U/L (*n* = 119)	128	39–909	105 (43–409)	174 (39–909)	<0.001
PSA μg/L (*n* = 120)	152	4.5–5300	120 (45–2000)	175 (7–5300)	0.519
CTC count (*n* = 114)	15.5	0–1025	1 (0–4)	51 (5–1025)	

Patients characteristics of the 120 patients included in this study. Note: CTC = circulating tumor cells, LHRH agonist = luteinizing hormone-releasing hormone agonist; mCRPC = metastatic castration-resistant prostate cancer; ALP = alkaline phosphatase; LDH = lactate dehydrogenase; PSA = prostate specific antigen. * One patient received enzalutamide and orteronel sequentially. CTC counts are from baseline CTC enumeration.

**Table 2:** Cox Regression analysis (*n* = 63).

(A)	Univariate Analysis			Multivariate Analysis		
Variable	HR	95% CI	*p*-Value	HR	95% CI	*p*-Value
5-gene profile	1.325	0.80–2.20	0.279			
CTC count	1.001	1.00–1.00	0.011	1.001	1.00–1.00	0.023
Age	0.988	0.95–1.02	0.508			
PSA	1.000	1.00–1.00	0.777			
ALP	1.001	1.00–1.00	0.101			
LDH	1.000	0.99–1.00	0.248			
Albumin	0.954	0.91–1.00	0.070			
Hemoglobin	0.709	0.54–0.93	0.011	0.731	0.56–0.95	0.020
**(B)**	**Univariate Analysis**			**Multivariate Analysis**		
**Variable**	**HR**	**95% CI**	***p*-Value**	**HR**	**95%CI**	***p*-Value**
5-gene profile	1.743	1.02–2.98	0.042			
CTC count	1.002	1.00–1.00	0.006	1.002	1.00–1.00	0.009
Age	1.015	0.98–1.05	0.418			
PSA	1.000	1.00–1.00	0.090			
ALP	1.001	1.00–1.00	0.038			
LDH	1.001	1.00–1.00	0.006			
Albumin	0.949	0.90–1.00	0.056			
Hemoglobin	0.631	0.48–0.84	0.002	0.642	0.48–0.85	0.002

Cox regression analysis in 63 patients with baseline gene expression data available for (A) progression-free survival and (B) overall survival. The 5-gene profile was dichotomized to high and low predicted risk, while the other variables are continuous. Note: ALP = alkaline phosphatase; HR = hazard ratio; LDH = lactate dehydrogenase; PSA = prostate specific antigen. CTC counts are from baseline CTC enumeration.

**Table 3:** Cox Regression analysis (*n* = 114).

(A)	Univariate Analysis			Multivariate Analysis		
Variable	HR	95% CI	*p*-Value	HR	95%CI	*p*-Value
CTC count	1.002	1.00–1.00	<0.001	1.002	1.00–1.00	<0.001
Age	0.989	0.96–1.02	0.453			
PSA	1.000	1.00–1.00	0.732			
ALP	1.002	1.00–1.00	<0.001	1.002	1.00–1.00	0.014
LDH	1.001	1.00–1.00	0.007			
Albumin	0.983	0.94–1.03	0.490			
Hemoglobin	0.694	0.57–0.85	<0.001	0.749	0.61–0.92	0.005
**(B)**	**Univariate Analysis**			**Multivariate Analysis**		
**Variable**	**HR**	**95% CI**	***p*-Value**	**HR**	**95%CI**	***p*-Value**
CTC count	1.003	1.00–1.00	<0.001	1.002	1.00–1.00	<0.001
Age	1.017	0.99–1.05	0.276			
PSA	1.000	1.00–1.00	0.112			
ALP	1.002	1.00–1.00	<0.001	1.002	1.00–1.00	0.006
LDH	1.002	1.00–1.00	<0.001			
Albumin	0.952	0.91–0.99	0.044			
Hemoglobin	0.551	0.44–0.68	<0.001	0.591	0.47–0.74	<0.001

Cox regression analysis in 114 patients with baseline CTC enumeration available for (A) progression-free survival and (B) overall survival. All variables are continuous in this regression analysis. Note: ALP = alkaline phosphatase; HR = hazard ratio; LDH = lactate dehydrogenase; PSA = prostate specific antigen. CTC counts are from baseline CTC enumeration.

## Supplementary Materials

The following are available online at https://www.mdpi.com/2072-6694/11/8/1212/s1: Figure S1: Clustering of gene expression data, Figure S2: PFS and OS in relation to the clustering groups, Figure S3: PFS and OS in the 5-gene expression profile at baseline, Figure S4: Cut off epithelial profile, Table S1: Genes used for gene-expression profile, Table S2: Univariate regression analysis PFS.
