# Supplementary material for: Circulating Tumor Cell Enumeration and Characterization in Metastatic Castration-Resistant Prostate Cancer Patients Treated with Cabazitaxel
Source: Cancers (Basel). 2019 Aug 20;11(8):1212. doi: 10.3390/cancers11081212 (PMC6721462; doi:10.3390/cancers11081212)
Supplement: Supplementary file 1 [file cancers-11-01212-s001.zip › Supp Table S1.pdf]

<u>Supplementary table S1.</u> Genes used for gene-expression profile

A	All 96 genes in the gene-expression panel (including 3 reference genes)			
ABCC4	*CDK12*	*FZD7*	*NTS*	*STYK1*
ACPP	*CDK4*	*GUCY1A3*	*PCGEM1*	*TACC2*
ADRA2C	*CEACAM5*	*HNF1A*	*PIK3CA*	*TBX3*
AGR2	*COL18A1*	*IGFBP4*	*PIK3CB*	*TCF7L2*
AKR1C3	*CREB3L4*	*IGFBP5*	*PLAU*	*TMPRSS2*
APC	*CRISP3*	*ITGA6*	*PMEPA1*	*TMPRSS2_ERG*
APP	*CYP11A1*	*KIT*	*PPAP2A*	*UGT2B15*
AR	*CYP17A1*	*KLK2*	*PPARG*	*WNT11*
AR_V1	*DDC*	*KLK3*	*PRKACB*	*WNT4*
AR_V3	*DHCR24*	*KRT8*	*PTEN*	*WNT5A*
AR_V7	*DKK1*	*LAD1*	*PTPN1*	*ZBTB16*
AR_V9	*DRG1*	*LEF1*	*RAD51B*	
AR_WT_fl	*EAF2*	*LRIG1*	*RAD51C*	Reference genes
AXIN2	*ELL2*	*MALAT1*	*RB1*	*GUSB*
BMP7	*EPCAM*	*MED12*	*S100A16*	*HMBS*
BRCA2	*EZH2*	*MKI67*	*SLC45A3*	*HPRT1*
CAV1	*FAT1*	*MSMB*	*SLCO1B3*	
CCND1	*FKBP5*	*MYC*	*SOX9*	
CDH1	*FOLH1*	*NDRG1*	*SP5*	
CDH10	*FOXA1*	*NKX3_1*	*SRD5A2*	

B	CTC-specific genes			
AGR2	*BMP7*	*FKBP5*	*MSMB*	*TACC2*
AR	*CCND1*	*FOLH1*	*NKX3_1*	*TBX3*
AR-V1	*CDH1*	*FOXA1*	*PPAP2A*	*TMPRSS2*
AR-V3	*CRISP3*	*KLK2*	*PPARG*	*TMPRSS2_ERG*
AR-V7	*DKK1*	*KLK3*	*SOX9*	*UGT2B15*
AR-V9	*EPCAM*	*KRT8*	*SP5*	*WNT5A*
AR_WT_fl	*FAT1*	*LAD1*	*STYK1*	

A) All 96 genes that were measured in the samples with available RNA. B) The 34 genes with higher expression in CTC samples compared to the background of leukocytes.
