# Supplementary material for: Circulating Tumor Cell Enumeration and Characterization in Metastatic Castration-Resistant Prostate Cancer Patients Treated with Cabazitaxel
Source: Cancers (Basel). 2019 Aug 20;11(8):1212. doi: 10.3390/cancers11081212 (PMC6721462; doi:10.3390/cancers11081212)
Supplement: Supplementary file 1 [file cancers-11-01212-s001.zip › Supp Table S2.pdf]

Supplementary table S2. Univariate regression analysis of PFS

Univariate analysis PFS

Gene	Exp(B)	95% CI	sig
AGR2	1.087	1.02-1.16	0.008
AR	1.069	1.0-1.15	0.059
AR_V1	1.012	0.96-1.07	0.653
AR_V3	1.034	0.96-1.12	0.393
AR_V7	1.013	0.93-1.10	0.768
AR_V9	0.996	0.94-1.06	0.905
AR_WT_fl	1.066	1.00-1.14	0.071
BMP7	1.042	0.99-1.10	0.141
CCND1	1.031	0.92-1.16	0.607
CDH1	1.037	0.96-1.13	0.391
CRISP3	0.962	0.86-1.08	0.498
DKK1	1.072	1.01-1.14	0.022
EPCAM	1.056	0.94-1.18	0.341
FAT1	1.068	1.01-1.13	0.030
FKBP5	1.004	0.84-1.21	0.969
FOLH1	1.047	1.00-1.10	0.074
FOXA1	1.078	1.01-1.15	0.024
KLK2	1.055	0.99-1.13	0.115
KLK3	1.034	0.97-1.10	0.310
KRT8	1.065	0.96-1.18	0.223
LAD1	1.036	0.99-1.09	0.146
MSMB	1.028	0.98-1.08	0.261
NKX3_1	1.012	0.78-1.31	0.928
PPAP2A	1.058	0.93-1.20	0.376
PPARG	0.985	0.85-1.14	0.832
SOX9	1.030	0.98-1.09	0.283
SP5	1.041	0.99-1.10	0.115
STYK1	1.013	0.95-1.08	0.699
TACC2	1.040	0.99-1.09	0.129
TBX3	1.050	0.99-1.11	0.092
TMPRSS2	1.058	1.00-1.12	0.037
TMPRSS2_ERG	1.026	0.98-1.08	0.318
UGT2B15	1.052	0.98-1.13	0.173
WNT5A	1.033	0.96-1.12	0.407

Univariate Cox Regression analysis of all 34 genes in comparison with PFS in 63 patients with available gene expression data.
