# Supplementary material for: Circulating Tumor Cell Enumeration and Characterization in Metastatic Castration-Resistant Prostate Cancer Patients Treated with Cabazitaxel
Source: Cancers (Basel). 2019 Aug 20;11(8):1212. doi: 10.3390/cancers11081212 (PMC6721462; doi:10.3390/cancers11081212)
Supplement: Supplementary file 1 [file cancers-11-01212-s001.zip › Supp Figures and Tables titles and captions.docx]

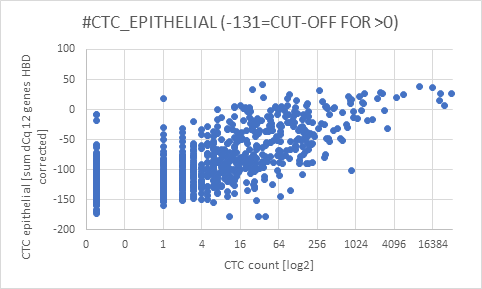

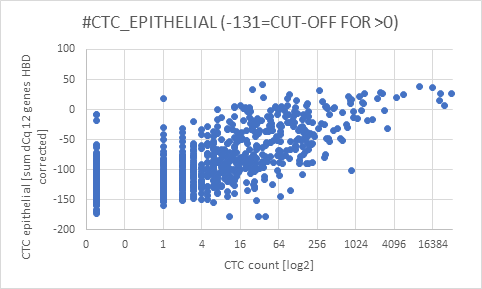
**Supplementary Figure S1**. Clustering of gene expression data

*Hierarchical clustering of the 34 CTC-specific genes in the 63 mCRPC patients. Red indicates above median and green below median expression level of a gene over all samples.*

**Supplementary Figure S2.** PFS and OS in relation to the clustering groups

*Kaplan-Meier curves of (A) progression-free survival (PFS) and (B) overall survival (OS) in relation to the groups divided by the cluster that was created in Supplementary Figure S1.*

**Supplementary Figure S3.** PFS and OS in the 5-gene expression profile at baseline

*Kaplan-Meier curves of (A) progression-free survival (PFS) and (B) overall survival (OS) in relation to the 5-gene expression profile. The groups are divided into two categories based on their low (1) and high (2) predicted risk based on this 5-gene profile by BRB Array tools.*

**Supplementary Figure S4**. Cut off epithelial profile

*Pearson correlation between the epithelial profile that has been determined on 12 genes in metastatic breast cancer patients and the epithelial profile used in this metastatic prostate cancer group based on 6 out of those 12 genes (A). In B) a receiving operating characteristic (ROC) curve with an area under the curve (AUC) value of this threshold is shown.*

**Supplementary table S1.** Genes used for gene-expression profile

*(A) All 96 genes that were measured in the samples with available RNA. (B) The 34 genes with higher expression in CTC samples compared to the background of leukocytes.*

**Supplementary Table S2**. Univariate regression analysis of PFS

*Univariate Cox Regression analysis of all 34 genes in comparison with PFS in 63 patients with available gene expression data.*
