# Supplementary figures and images for: Circulating Tumor Cell Enumeration and Characterization in Metastatic Castration-Resistant Prostate Cancer Patients Treated with Cabazitaxel
Source: Cancers (Basel). 2019 Aug 20;11(8):1212. doi: 10.3390/cancers11081212 (PMC6721462; doi:10.3390/cancers11081212)

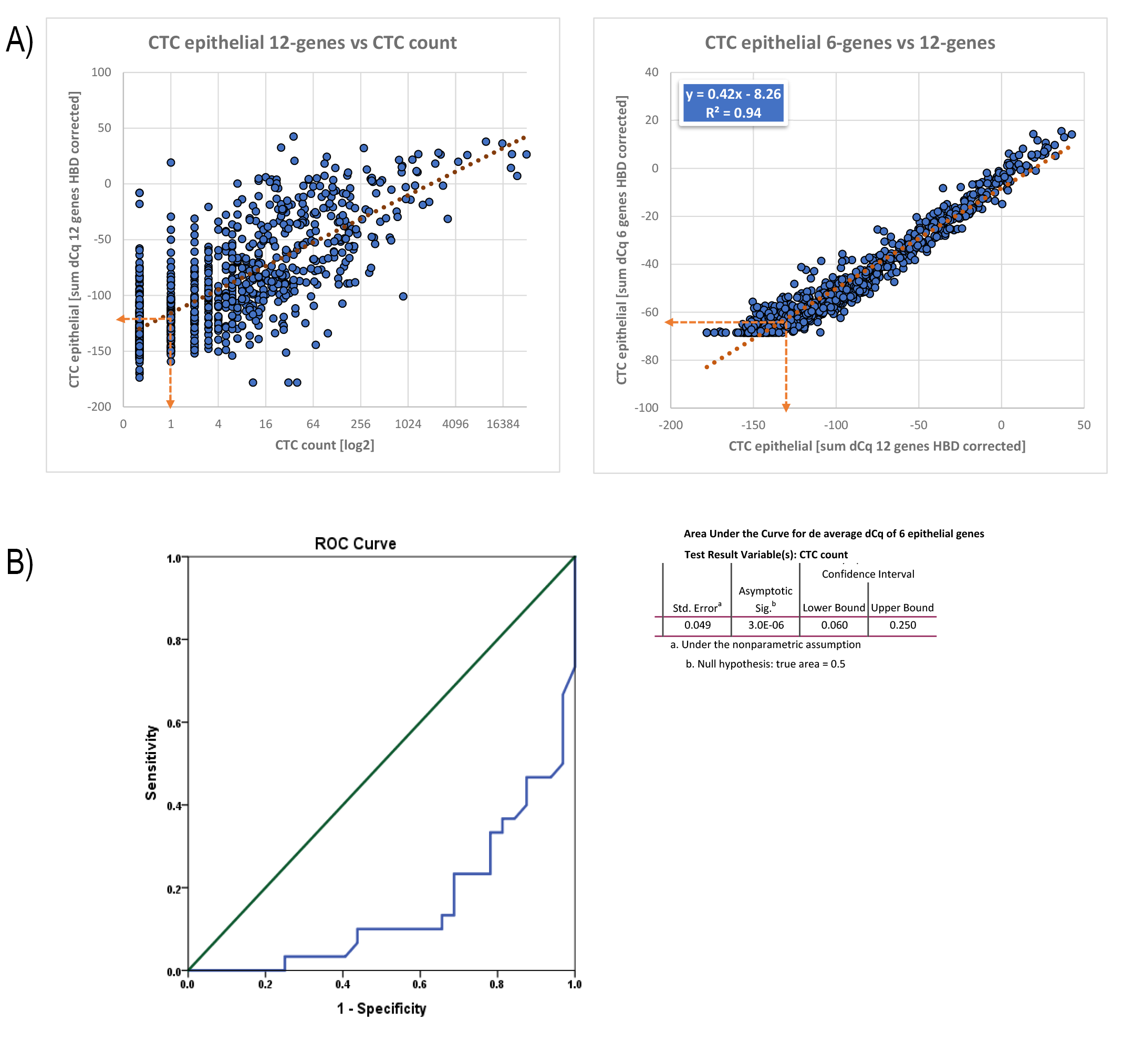

Supplement: Supplementary file 1 [file cancers-11-01212-s001.zip › Supp Figure 4 - Epithelial profile.tif]

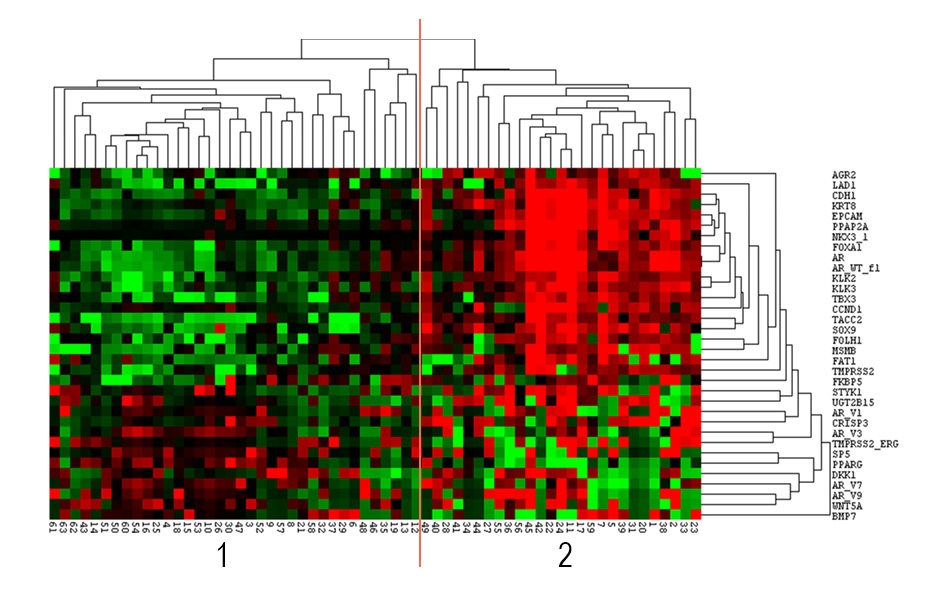

Supplement: Supplementary file 1 [file cancers-11-01212-s001.zip › Supp Figure 1 - Cluster dd 15-07-2019.tif]

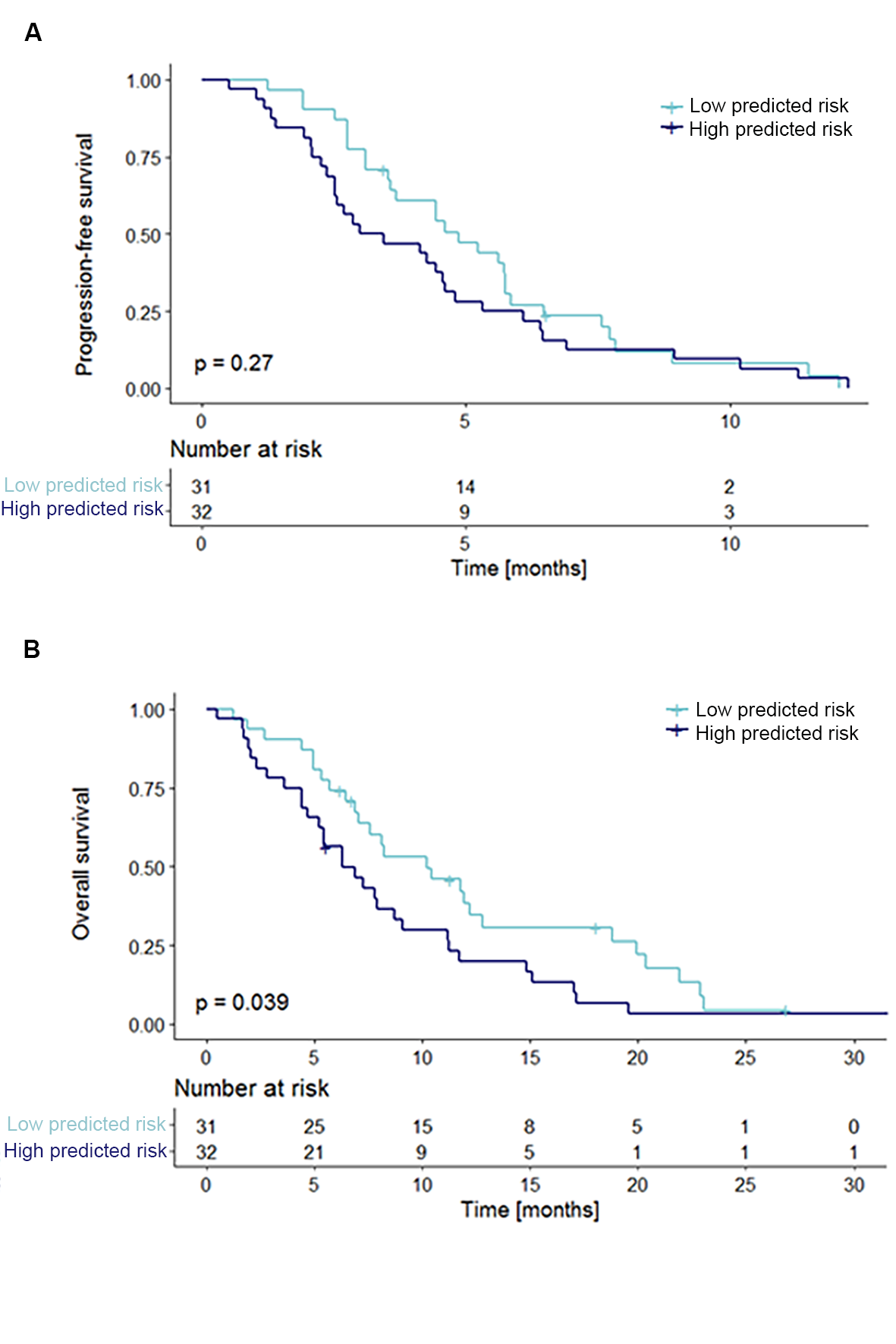

Supplement: Supplementary file 1 [file cancers-11-01212-s001.zip › Supp Figure 3 - OS and PFS vs Risk preds2.tif]

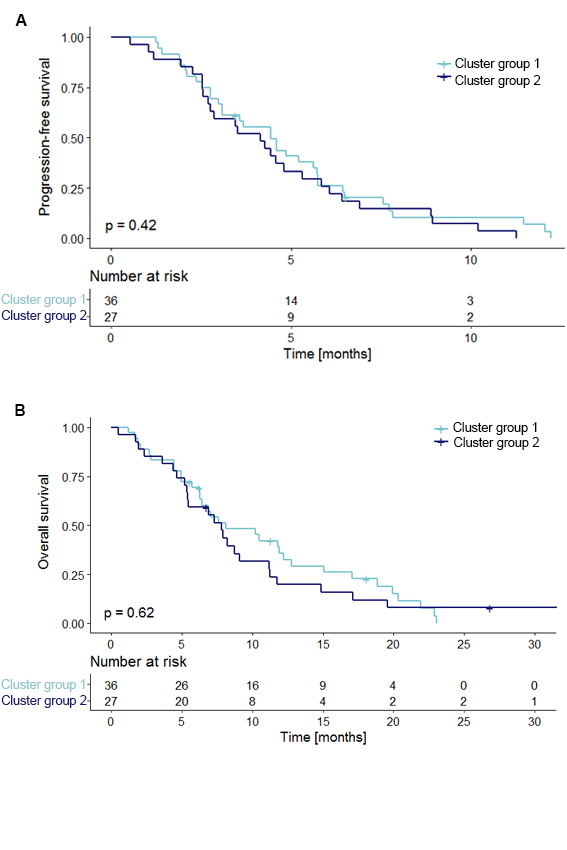

Supplement: Supplementary file 1 [file cancers-11-01212-s001.zip › Supp Figure 2 - PFS and OS cluster dd 15072019.tif]
